# Editorial: Fetal phenotypes of rare diseases: application and evaluation of prenatal exome sequencing and pathogenesis research of rare diseases

**DOI:** 10.3389/fgene.2023.1205726

**Published:** 2023-05-09

**Authors:** Yan Zhang, Can Ding, Yiwei Chen, Meng Wu, Min Luo

**Affiliations:** ^1^ Center for Medical Genetics, Guangdong Women and Children Hospital, Guangzhou, China; ^2^ Universitätsmedizin Marburg—Campus Fulda, Fulda, Germany; ^3^ Wonder Sir, Shanghai, China; ^4^ Shandong Provincial Key Laboratory of Radiation Oncology, Shandong Cancer Hospital and Institute, Shandong First Medical University and Shandong Academy of Medical Sciences, Jinan, China; ^5^ Department of Biological Sciences, National University of Singapore, Singapore, Singapore

**Keywords:** exome sequencing, phenotype [mesh], prenetal diagnosis, fetal abnormalities, rare disease (RD)

The use of exome sequencing in prenatal diagnosis is an exciting development in the field of medical genetics. However, there are still challenges and limitations to be addressed. One challenge is the difficulty in obtaining reliable fetal phenotypes for many genetic disorders. Additionally, the prenatal pathogenesis of many diseases remains unknown. This Research Topic aims to address these challenges by compiling cases of fetal abnormalities and meticulously documenting both prenatal and postnatal phenotypes to broaden our understanding of specific genetic diseases and their pathogenesis. The value of exome sequencing in diagnosing fetuses with structural abnormalities will be evaluated by combining it with traditional prenatal diagnosis technology.

This Research Topic explores three types of common fetal abnormalities: neurologic abnormalities, skeletal anomalies, and congenital heart diseases. Joubert syndrome (JBS), a neurodevelopmental disorder characterized by a distinctive malformation of the brainstem, has been called the “molar tooth sign” on ultrasound screening or MRI imaging. Individuals with JBS may have a variety of symptoms including breathing abnormalities, delayed development, abnormal eye movements, and kidney, liver, and retinal abnormalities. The severity of the condition can vary widely from person to person, even within families. The prenatal diagnosis of JBS has always been tricky. Li et al. used whole-exome sequencing and other techniques to identify a causative variation in the *OFD1* gene in a suspected JBS family. While Huang et al. analyzed the relationship between genotypes and prenatal imaging phenotypes in 13 fetuses with JBS. The molar sign was found by MRI in 10 fetuses while ultrasound in 11 fetuses. Pathogenic/likely pathogenic variations in *OFD1, TMEM67, CC2D2A, RPGRIP1L, TCTN3, CEP290, NPHP1* genes were detected with exome sequencing in the 13 fetuses. Other than the molar sign, distinct prenatal imaging phenotypes were showed with MRI or ultrasound in the fetus with different causative genes.

Abnormal fetal short long bones are frequently detected during prenatal sonographic examinations, and may be associated with various genetic disorders, such as skeletal dysplasia, achondroplasia, and other Mendelian disorders. Huang et al. analyzed 94 fetuses with short long bones found by routing prenatal sonographic examine. Exome sequencing detected causative pathogenic variants in 40.4%. Achondroplasia, osteogenesis imperfecta, thanatophoric dysplasia, chondrogenesis and 3-M syndrome were the most common associated Mendelian disorders. Hence they recommend genetic testing for fetuses with femur length shorter than -4SDs of gestational age.

Congenital heart defects (CHDs) are another kind of common birth defect, and identifying biomarkers for prenatal diagnosis can be challenging due to their complex nature. Liu et al. conducted a study to identify epigenetic biomarkers for conotruncal heart defects (CTDs), and found that methylation levels in placental tissue can differ significantly between fetuses with CTDs and the control. The study suggests that epigenetic biomarkers such as *HOXD9, CNN1, NOTCH1,* and *ECE1* could be potential candidates in cell-free fetal DNA tests to predict fetuses with CTDs.

As genome technologies become increasingly integrated into clinical practices, it is crucial to establish reliable and robust application standards for prenatal diagnosis. Unlike pediatric cases, prenatal cases require predictive testing to determine whether the fetus has a serious genetic disease based on the test results, which can inform decisions on whether to proceed with the pregnancy. However, challenges remain in interpreting the vast number of variations of unknown significance and obtaining detailed fetal phenotypes in a timely manner. Therefore, further research is required to refine and standardize the use of genome technologies, such as exome sequencing, in prenatal diagnosis. This will enable healthcare professionals to accurately diagnose and counsel parents on the potential risks and outcomes of their pregnancy, ultimately leading to better informed decision-making and improved patient care.

